# Demographic and Socioeconomic Correlates of Disproportionate Beef Consumption among US Adults in an Age of Global Warming

**DOI:** 10.3390/nu15173795

**Published:** 2023-08-30

**Authors:** Amelia Willits-Smith, Harmonii Odinga, Keelia O’Malley, Donald Rose

**Affiliations:** 1 Tulane Nutrition, School of Public Health & Tropical Medicine, Tulane University, New Orleans, LA 70112, USA; ameliaws@unc.edu (A.W.-S.); odinga@livemail.uthscsa.edu (H.O.); komalley@tulane.edu (K.O.); 2 Global Food Research Program, University of North Carolina, Chapel Hill, NC 27516, USA; 3 Long School of Medicine, University of Texas Health Science Center, San Antonio, TX 78229, USA

**Keywords:** NHANES, diet recall, beef intake, MyPlate, climate change

## Abstract

Concern for the environment when making dietary choices has grown as the contribution of the food sector to global greenhouse gas emissions becomes more widely known. Understanding the correlates of beef eating could assist in the targeting of campaigns to reduce the consumption of high-impact foods. The objective of this study was to identify the demographic, socioeconomic, and behavioral correlates of disproportionate beef consumption in the United States. We analyzed 24-h dietary recall data from adults (*n* = 10,248) in the 2015–2018 National Health and Nutrition Examination Survey (NHANES). Disproportionate beef consumption was defined as an intake greater than four ounce-equivalents per 2200 kcal. Associations of this indicator variable with gender, age, race/ethnicity, education, family income, diet knowledge, and away-from-home meals were assessed using logistic regression, incorporating survey design and weighting. Disproportionate beef diets were consumed by 12% of individuals, but accounted for half of all beef consumed. Males were more likely than females (*p* < 0.001) to consume these diets. This relationship was seen in all bivariate and multivariable models. Older adults, college graduates, and those who looked up the MyPlate educational campaign online were less likely (*p* < 0.01) to consume a disproportionate beef diet. While almost one-third of reported consumption came from cuts of beef (e.g., steak or brisket), six of the top ten beef sources were mixed dishes: burgers, meat mixed dishes, burritos and tacos, frankfurters, soups, and pasta. Efforts to address climate change through diet modification could benefit from targeting campaigns to the highest consumers of beef, as their consumption accounts for half of all beef consumed.

## 1. Introduction

The dietary habits of Americans need improving. Assessed with data from recent national surveys, the US diet scores only 59 points on the 100-point Healthy Eating Index (HEI) scale, an aggregate measure of consumption designed to reflect the latest Dietary Guidelines for Americans (DGA) [1]. The HEI is broken down into 13 components, and US diets score particularly low on whole grains, sodium, fatty acids, and saturated fats.

Part of the explanation for the low scores on the latter three of these components comes from excess meat consumption of some population subgroups. On average, teenage boys consume more meat, poultry, and eggs than is recommended in the DGA [2]. For adult men, the distance from the recommendations is even greater.

In addition to health concerns, excess meat consumption has serious environmental impacts [3]. Numerous studies have documented our collective impact on climate change, with the food sector playing a big role; recent estimates indicate that about one-third of greenhouse gas emissions (GHGE) are due to human food systems [4]. Meat, particularly from ruminant animals, is at the top of the list of impactful foods. Livestock alone accounts for 14% of global GHGE [5].

There is wide variation in GHGE across types of meat. For example, on an edible portion weight basis, beef is about eight times more impactful than chicken and six times more so than pork [6]. The main reason for this differential impact is that ruminant animals emit far more methane than monogastric animals, that is, those with just one stomach. Methane is a potent greenhouse gas that has over 25 times the global warming potential of carbon dioxide [7]. Because of their potency, a recent United Nations report indicated that reducing methane emissions is one of the most cost-effective strategies to rapidly reduce the rate of global warming [8].

A number of national and international dietary recommendations now consider the sustainability of the food supply along with nutritional criteria, and have promoted a reduction in meat consumption in high-income countries. This includes the EAT-Lancet recommendations, as well as those developed for the Netherlands, Sweden, Germany, and others [9,10,11]. In the US, an earlier panel of experts recommended a diet higher in plant-based foods and lower in animal-based foods, although this was never adopted by the government in the publication of their DGA [12]. Evidence suggests that providing consumers with information about the environmental impacts of meat consumption reduces the intention to eat meat, but more work is needed to see whether these intentions translate to reduced consumption [13].

Programmatic attempts to reduce meat consumption would benefit from a greater understanding of the problem. In particular, those targeting promotional or educational campaigns need to know in which population segments consumption is high. Previous research seems to indicate that consumption is higher among men, Hispanics, middle-aged adults, and those with less education [14,15,16,17]. However, the results vary by study and by type of meat. Although a number of studies have focused on red meat [14,15], few of them have focused on beef, per se [16,17]. The importance of being clear about the source of meat (say, beef vs. pork vs. poultry) is important for environmental reasons, as summarized above and indicated previously [18]. Moreover, all of these studies either make comparisons of mean consumption or compare consumption versus not consuming; they have not identified the characteristics of those with high consumption levels. This would be useful for targeting those with the greatest impact on both health and the environment. To address this need, we evaluated the demographic and socioeconomic characteristics associated with disproportionate beef consumption in the US.

## 2. Materials and Methods

### 2.1. Study Sample

This study was conducted using the 2015–2018 National Health and Nutrition Examination Survey (NHANES), a nationally representative survey of the civilian, non-institutionalized population living in the US. All adults (*n* = 10,248), 18 years and older, that had reliable dietary data were included in our analytic sample. See below for additional details of the diet methodology. Details about the NHANES sample design have been published previously [19].

### 2.2. Dietary Data: Calculating Outcome Variable

Dietary data were collected in the NHANES using an automated 24-hour recall instrument administered by trained enumerators. We focused on the first day of dietary intake, which was administered in the NHANES Mobile Examination Center [20]. Dietary data go through a number of checks, and those recalls that were considered reliable and met minimum acceptability criteria by the survey team were included in this analysis [21].

The main focus of this research was to identify the characteristics of disproportionate beef consumers. However, there is no simple “beef” variable in the NHANES databases. Unlike commodity data sources, the 24-hour dietary recall data are composed of “as-consumed” foods, such as lasagna or burritos, which may contain beef, as well as many other items. Using the weight of lasagna consumed, for example, would not be an accurate measure of the amount of beef consumed.

To address this issue, we employed the Food Patterns Equivalents Database (FPED), which is developed in conjunction with the NHANES [22]. The FPED translates the amounts of the NHANES “as-consumed” foods into corresponding amounts of the nutritional food groups used in scoring the Health Eating Index. All of the beef in the as-consumed food item amounts was translated to ounce-equivalents (representing the lean fraction) of protein foods and placed into one of the following three food groups: (1) Beef, veal, pork, lamb, and game meat; excluding organ meats and cured meat; (2) Cured/luncheon meat made from beef, pork, or poultry; or (3) Organ meat from beef, veal, pork, lamb, game, and poultry.

In order to account for just the beef amounts in these food groups, we used this information in combination with text from the food description for each item in order to weight how much of the ounce equivalents came from beef. If the description said the food contained beef (e.g., “Beef burrito”), but mentioned no other meat, then the full amount of ounce equivalents from the above-bulleted groups was allocated to beef. If the food description included beef alongside another meat (e.g., “Frankfurter with beef and pork”) or just referred to meat (e.g., “lasagna with meat”), we allocated half of the ounce equivalents from the above groups to beef. Confirmation of these choices was made by looking up recipes in the USDA’s FoodData Central [23] and occasionally by using Google for common recipes (e.g., mortadella).

Intake amounts from these three FPED groups were summed for each individual. Individuals were then classified as “disproportionate beef eaters” if they consumed more than four ounce-equivalents per 2200 kcal. In the DGA, the “Healthy US-Style Dietary Pattern” for a 2200 kcal level recommends 4 oz eq/d for all meats, poultry, and egg products combined [2], so we considered an amount above this threshold from just beef alone to be disproportionate. Scaling was important since the energy requirements of individuals vary, and we used the 2200 kcal diet plan since that roughly corresponds to the mean energy intake of our sample.

### 2.3. Demographic, Socioeconomic, and Behavioral Data: Predictor Variables

Basic demographic variables were used to describe individuals. These included gender, age, race/ethnicity, educational level, family income, and family size. The NHANES variable labeled “Gender of the participant” is collected using the following instructions: “ASK IF NOT OBVIOUS: Is {NAME} male or female?” While the answer options seem to reflect sex assigned at birth, we followed the NHANES codebook and called the variable gender. Age was divided into four categories: 18–29, 30–49, 50–65, and 66 years and older. Race/ethnicity was self-identified and grouped by the NHANES into the following categories: Mexican Americans, other Hispanics, non-Hispanic Whites, non-Hispanic Blacks, non-Hispanic Asians, and those of other or multiple races/ethnicities. Education included those with less than a high school education, high school graduates or equivalent, those with some college, and college graduates. The income to poverty ratio (IPR, which equals family income divided by the poverty threshold) was categorized into those with an IPR < 1 (i.e., in poverty), 1–1.99, 2–4.99, and 5 and over. We also categorized family size: 1, 2, 3–4, and 5 and over.

Behavioral characteristics included whether or not the respondent had heard of the MyPlate educational campaign, whether they looked it up on the Internet, and whether or not they tried it. Respondents were also asked to evaluate the healthiness of their diet, which included five categories ranging from poor to excellent. The number of meals prepared away from home in the last seven days was divided into two groups: those consuming five or more meals prepared away from home and those consuming fewer than that. Respondents were asked about how often they ate ready-to-eat foods from grocery stores (e.g., salads, soups, chicken, sandwiches, and cooked vegetables from salad bars and deli counters) in the last 30 days. They were also asked about the number of frozen meals or frozen pizza eaten in the last 30 days. We categorized both of these into groups of individuals consuming ten or more of the foods per month.

### 2.4. Statistical Analysis

Dietary sample weights and survey design parameters included with the NHANES datasets were used for all of the results presented here [19]. Bivariate logistic regressions were used to test for associations of demographic, socioeconomic, and behavioral variables individually with disproportionate beef consumption. We examined three multivariable logistic regression models of increasing complexity, starting with demographic predictors, and then adding socioeconomic and behavioral predictors in succession. Family size was not included in multivariable models, because it is an element of the poverty threshold used to calculate the income-to-poverty ratio. Looking up MyPlate guidance was chosen as the behavioral predictor for the multivariable models since it indicated an actual action, compared to simply hearing about MyPlate. Tests with *p*-values < 0.05 were considered statistically significant. Analyses were run in Stata standard edition version 17.0.

## 3. Results

Table 1 displays the demographic and socioeconomic characteristics of our analytic sample as well as the associations of these characteristics and disproportionate beef consumption. The NHANES sample is representative of the US non-institutionalized population, with about half being women, a third being individuals aged 30–49 years, over 60% being non-Hispanic White, and over 60% having some college education or more. About 13% of individuals are in poverty and 22% live alone (percentages not shown).

**Table 1:** Demographic characteristics of disproportionate beef consumers.

		Disproportionate Beef Consumption (>4 oz/2200 kcal)				
	*n*	No (%)	Yes (%)	OR	95%CI	*p*
**Total**	10,248	87.8	12.2			
**Gender**						
Male	4969	85.4	14.6	1.55	1.24, 1.93	**<0.001**
Female	5279	90.1	9.9	-------	-------	-------
**Age (Years)**						
18–29	2018	89.1	11.0	0.71	0.52, 0.97	**0.034**
30–49	3125	88.1	11.9	0.78	0.58, 1.04	0.091
50–65	2842	85.2	14.8	-------	-------	-------
66–80+	2263	89.7	10.3	0.66	0.45, 0.97	**0.034**
**Race/Ethnicity**						
Non-Hispanic White	3535	87.4	12.6	-------	-------	-------
Non-Hispanic Black	2293	90.4	9.6	0.74	0.58, 0.94	**0.016**
Mexican American	1612	86.2	13.8	1.11	0.89, 1.38	0.336
Other Hispanic	1147	87.0	13.0	1.03	0.80, 1.33	0.787
Non-Hispanic Asian	1205	91.8	8.2	0.62	0.48, 0.80	**0.001**
Other, incl. multiracial	456	85.9	14.1	1.14	0.77, 1.70	0.497
**Education**						
<High school	2185	87.4	12.6	0.86	0.59, 1.25	0.407
High school graduate	2487	85.7	14.4	-------	-------	-------
Some college	3159	87.5	12.5	0.85	0.64, 1.14	0.272
College graduate	2407	90.1	9.9	0.65	0.48, 0.89	**0.007**
**IPR**						
<1	1902	89.2	10.9	0.93	0.74, 1.17	0.504
1–<2	2521	88.4	11.6	-------	-------	-------
2–<5	3194	88.0	12.0	1.03	0.79, 1.35	0.798
5+	1528	87.1	12.9	1.12	0.85, 1.48	0.396
Missing	1103	85.5	14.5	1.29	0.91, 1.84	0.148
**Family Size**						
1	2151	88.0	12.0	0.94	0.74, 1.19	0.600
2	2584	88.0	12.0	0.94	0.73, 1.22	0.656
3–4	3274	87.3	12.7	-------	-------	-------
5+	2239	88.1	11.9	0.93	0.69, 1.25	0.623

**Notes:** Data are from adults aged 18+ years with reliable day 1 diet recalls in the 2015–2018 US NHANES. OR, odds ratio from bivariate logistic regression; -------, reference group; and IPR, income to poverty ratio, i.e., family income divided by the poverty threshold. Bold values are statistically significant at *p* < 0.05.

Overall, 12.2% of adults were classified as disproportionate beef consumers (>4 oz-eq/2200 kcal). In bivariate logistic regression models, disproportionate beef consumption was significantly associated with gender; males were 1.55 times (95% CI 1.24, 1.93) more likely to be disproportionate beef consumers than females. Disproportionate beef consumption ranged across race/ethnicity categories, from 8.2% for non-Hispanic Asians to 14.1% for those who were other/multiracial. Non-Hispanic Blacks and Asians had significantly lower odds of being a disproportionate beef consumer than non-Hispanic Whites, as did those aged 66 years and older, or those 18–29 years, compared to those that were 50–65 years of age. College graduates were less likely to be disproportionate beef eaters than high school graduates. Disproportionate beef consumption was not significantly associated with family income or family size.

For behavioral correlates of disproportionate beef consumption (Table 2), those who had heard of MyPlate or looked up MyPlate online were less likely to be disproportionate beef consumers. Other behavioral characteristics were not significantly associated with disproportionate beef consumption.

**Table 2:** Behavioral characteristics of disproportionate beef consumers.

		Disproportionate Beef Consumption (>4 oz/2200 kcal)				
	*n*	No (%)	Yes (%)	OR	95%CI	*p*
**Total**	10,248	87.8	12.2			
**Heard of MyPlate**						
Yes	2055	90.8	9.2	0.67	0.52, 0.86	**0.003**
No	8193	86.8	13.2	-------	-------	-------
**Looked up MyPlate**						
Yes	769	92.9	7.1	0.53	0.36, 0.77	**0.002**
No	9479	87.3	12.7	-------	-------	-------
**Tried MyPlate**						
Yes	740	91.6	8.4	0.64	0.40, 1.02	0.058
No	9508	87.5	12.5	-------	-------	-------
**Diet Healthiness (Self-Assessed)**						
Excellent	783	90.0	10.1	0.76	0.48, 1.20	0.230
Very Good	1941	88.2	11.8	0.91	0.70, 1.20	0.503
Good	4062	87.5	12.5	0.97	0.80, 1.18	0.757
Fair	2726	87.2	12.8	-------	-------	-------
Poor	734	87.8	12.2	0.95	0.65, 1.37	0.758
**≥5 Away From Home Meals/Week**						
No	7812	87.9	12.1	-------	-------	-------
Yes	2424	87.7	12.3	1.01	0.81, 1.26	0.908
**≥10 Ready-to-Eat Meals/Week**						
No	9600	88.0	12.0	-------	-------	-------
Yes	611	85.4	14.6	1.25	0.80, 1.95	0.306
**≥10 Frozen Food or Pizza Meals/Week**						
No	9526	87.8	12.2	-------	-------	-------
Yes	705	87.4	12.6	1.04	0.74, 1.48	0.801

**Notes**: Data are from adults aged 18+ years with reliable day 1 diet recalls in the 2015–2018 US NHANES. OR, odds ratio from bivariate logistic regression; -------, reference group; and MyPlate, a food guide tool derived from the US Dietary Guidelines for Americans. Bold values are statistically significant at *p* < 0.05.

Results from our multivariable logistic regression analysis are presented in Table 3. Across all models, gender was the strongest predictor of disproportionate beef consumption. In the full model, males were 1.48 times (95% CI: 1.19, 1.84) more likely than females to have such diets. As in the bivariate results, non-Hispanic Blacks and Asians were less likely to have such diets compared to non-Hispanic Whites, as were college graduates compared to high school graduates. Those who looked up MyPlate food guidance were also less likely to consume disproportionate amounts of beef.

**Table 3:** Multivariable models predicting disproportionate beef consumption (>4 oz eq/2200 kcal).

	Model 1: Demographics			Model 2: Demographics + Socioeconomic			Model 3: Demographics + Socioeconomic + Behavioral		
	OR	95%CI	*p*	OR	95%CI	*p*	OR	95%CI	*p*
**Gender**									
Male	1.55	1.24, 1.93	**< 0.001**	1.52	1.23, 1.89	**< 0.001**	1.48	1.19, 1.84	**0.001**
Female	-------	-------	-------	-------	-------	-------	-------	-------	-------
**Age (Years)**									
18–29	0.70	0.51, 0.97	**0.031**	0.70	0.51, 0.96	**0.030**	0.72	0.53, 1.00	**0.048**
30–49	0.78	0.58, 1.04	0.088	0.82	0.62, 1.08	0.150	0.83	0.62, 1.09	0.171
50–65	-------	-------	-------	-------	-------	-------	-------	-------	-------
66–80+	0.66	0.44, 0.98	**0.039**	0.67	0.45, 1.00	**0.047**	0.65	0.44, 0.98	**0.041**
**Race/Ethnicity**									
Non-Hispanic White	-------	-------	-------	-------	-------	-------	-------	-------	-------
Non-Hispanic Black	0.74	0.58, 0.95	**0.019**	0.72	0.55, 0.94	**0.017**	0.72	0.55, 0.93	**0.015**
Mexican American	1.12	0.89, 1.41	0.317	1.08	0.82, 1.41	0.571	1.07	0.82, 1.40	0.584
Other Hispanic	1.04	0.81, 1.34	0.748	1.01	0.78, 1.31	0.916	1.00	0.77, 1.30	0.991
Non-Hispanic Asian	0.62	0.48, 0.81	**0.001**	0.67	0.51, 0.87	**0.005**	0.66	0.50, 0.86	**0.003**
Other, incl. multiracial	1.12	0.75, 1.67	0.575	1.14	0.75, 1.71	0.529	1.15	0.76, 1.72	0.502
**Education**									
< High school				0.86	0.57, 1.28	0.444	0.86	0.57, 1.28	0.444
High school graduate				-------	-------	-------	-------	-------	-------
Some college				0.84	0.64, 1.11	0.215	0.86	0.65, 1.14	0.275
College graduate				0.61	0.47, 0.80	**0.001**	0.65	0.49, 0.86	**0.004**
**IPR**									
<1				0.93	0.73, 1.18	0.534	0.93	0.73, 1.18	0.520
1–<2				-------	-------	-------	-------	-------	-------
2–<5				1.08	0.82, 1.43	0.566	1.08	0.82, 1.42	0.556
5+				1.24	0.94, 1.62	0.119	1.24	0.95, 1.62	0.115
Missing				1.41	0.97, 2.05	0.072	1.39	0.96, 2.02	0.078
**Looked up MyPlate**									
Yes							0.62	0.41, 0.95	**0.030**
No							-------	-------	-------
Model F value	4.502			2.954			3.137		
Model *p* value	0.0019			0.0210			0.0180		

**Notes**: Data are from adults aged 18+ years with reliable day 1 diet recalls in the 2015–2018 US NHANES (*n* = 10,248). OR, odds ratio from multivariable logistic regression including variables from the left-most column; -------, reference group; and MyPlate, a food guide tool derived from the US Dietary Guidelines for Americans. Bold values are statistically significant at *p* < 0.05.

**Figure 1:**
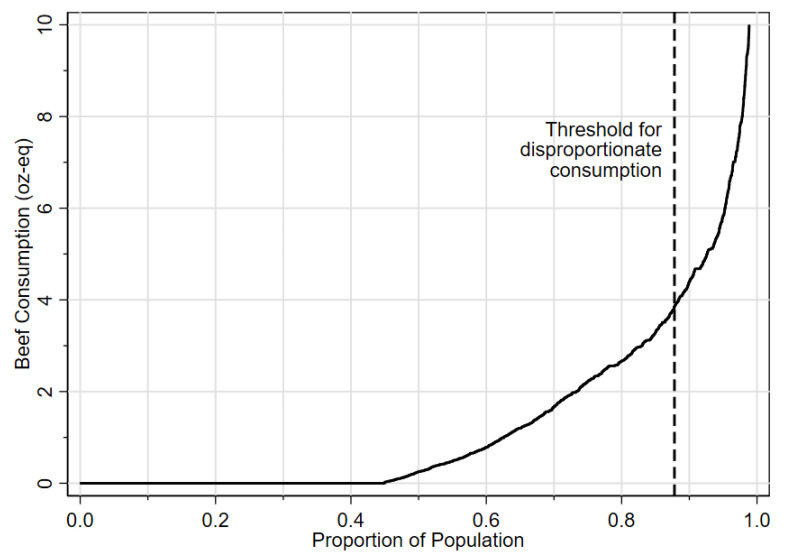
Cumulative distribution of beef consumed among US adults, day 1, NHANES 2015–2018. The area under the curve indicates the cumulative amount of beef consumed on the recall day for the proportion of the population at any given point.

Graphic results on the cumulative amount of beef consumption across the entire sample are presented in Figure 1, with the area under the curve being the cumulative amount at any given point. About 45% of the population had zero beef consumption on any given day, whereas the 12% of disproportionate beef consumers accounted for 50% of the total beef consumed.

Table 4 displays the top 20 sources of beef for all consumers of beef on the interview day, ranked in descending order by the total amount consumed, weighted to the population level. The largest source is a broad category that includes different cuts of beef (e.g., beef pot roast, beef short ribs, beef steak, beef brisket, etc.), which accounts for 31% of the total consumption. The top ten groups—which includes this general beef group, as well as burgers, meat mixed dishes (like meatloaf or beef stew), burritos and tacos, cold cuts and cured meats, ground beef, frankfurters, sausages, soups, and pasta mixed dishes (like spaghetti with meat sauce)—account for 89% of the total amount consumed. As for the frequency of consumption in the sample, the top five foods are beef, burgers, meat mixed dishes, burritos and tacos, and cold cuts and cured meats. Supplementary Materials Table S1 shows these results further broken down by age and gender.

## 4. Discussion

The strongest and most consistent predictor of disproportionate beef intake was gender. Men were more likely to do this, in both bivariate and multivariable models. In other bivariate results, the frequency of disproportionate beef consumers appeared to peak at 50–65 years (14.8%) and also among high school graduates (14.4%), and is lower among younger (18–29 years) and older (>65 years) consumers, college graduates, non-Hispanic Blacks, and non-Hispanic Asians. These associations remained significant in the multivariable models.

Although previous authors have studied meat or protein foods in the US population, their study aims were different than in this paper and so were their outcome measures. Still, our results are broadly consistent with previous literature, particularly for gender, education, age, and race/ethnicity. For example, An and colleagues, using NHANES data from 2005–2016, found that women, non-Hispanic African Americans, those 65 years and above, and those with some college education were less likely to eat beef (of any amount) [16]. Kim and colleagues studied trends in types of protein foods consumed from 1999 to 2010 using NHANES data, and found that mean beef intake amount was lower to begin with and decreased by more for college graduates than those with other levels of education [17]. Women, older adults, and those with more education had lower consumption levels of total meat (not just beef) in an earlier study using NHANES data from 1988 to 1994 [14]. Zeng et al. showed that the intake of unprocessed red meat was lower among women, older adults, non-Hispanic Blacks, and college graduates in the 2015–2016 NHANES [15].

While all of the studies mentioned above made important contributions, none of them evaluated disproportionate beef consumption or its correlates. We approached the issue differently and sought to understand the characteristics of those who are eating beef at the high end of the intake distribution. We also developed a novel approach to separate beef consumption from that of other red meat, making use of existing information available in national databases, like the FPED. At a time when climate change is an over-riding issue, the consumption of an environmentally extravagant source of protein is of concern, as is who to target with messaging about this problem. This becomes all the more important when considering another of our study’s findings: disproportionate beef consumers (>4 oz eq/2200 kcal) were responsible for half of all beef consumption on a given day.

Our threshold for disproportionate consumption was based on the total meat, poultry, and eggs amount recommended in the 2020–2025 Dietary Guidelines for Americans (DGA) Healthy US-Style Dietary Pattern: 4 oz-eq/day for those with a 2200 kcal diet. We considered consuming above this level to be disproportionate because such a recommendation could be met through a combination of different animal products, including pork, poultry, and eggs, not just beef. Protein food recommendations could even be met without any meat, poultry, or fish, using the DGA’s recommended Healthy Vegetarian Dietary Pattern [2]. Clearly, when we have an environmental crisis, and one food has eight times the impact of foods with similar nutritional benefits, it seems useful to develop some sort of threshold for what would be considered disproportionate. Our suggestion could be considered very generous, given that the upper end of the range for the EAT-Lancet Planetary Health Diet weekly limit for beef and lamb consumption scales to about 0.5 oz (14 g) per day, or about one-eighth of our threshold. There also seems to be utility in our threshold for targeting purposes, since disproportionate consumers accounted for 50% of all beef consumed.

As stated above, males were more likely to be disproportionate beef eaters than females. This may be because meat, especially red meat, is associated with masculinity, strength, and power in Western culture [24,25]. Men are more likely to subscribe to the idea that human lives are more valuable than those of animals, and are more likely to associate meat with healthiness [26,27]. Whatever the reasons, men are significantly less likely than women to consider reducing their meat consumption [28].

We also found that disproportionate beef consumption was inversely associated with knowledge about or actions on dietary guidance. Those who were not disproportionate beef eaters were more likely to have heard of the USDA’s MyPlate food guidance system or have looked it up online. This is potentially very important, because it might indicate that the DGA are a useful tool for promoting behavior change in this area. However, these results are cross-sectional; so, for example, we cannot assume that knowledge of MyPlate influenced meat-eating behavior. It may simply be that those more motivated about their diet reduced meat consumption and later looked into the government advice on diet. However, if even a small number of US consumers were influenced by dietary guidance to reduce beef consumption, the impacts on food-related emissions could be substantial [29]. Other dietary behaviors—such as the number of meals eaten away per week, or those that were ready-to-eat meals, frozen foods, or pizzas—were not associated with such disproportionate beef consumption.

Interestingly, we found that beef was consumed across a wide variety of foods, not just cuts of beef or burgers, but also in mixed dishes, including burritos, tacos, pasta, and sandwiches. This is consistent with the work of Frank and colleagues, who came to a similar conclusion in their study on US consumption of red and processed meats [30].

One limitation of this work is that it was based on 1-day diet recalls, so our results do not represent usual intake. Averaging both days of data available on the NHANES would not address this problem, would reduce our sample size by 15%, and would mix recall methods between an in-person interview (day 1) and one done on the phone (day 2). Still, as a check, we examined day 2 and found the same associations with gender and MyPlate guidance. Other associations were similar in magnitude, though not always significant. Another potential limitation is that the NHANES is a US study, and the data we analyzed are from 2015–2018. Thus, these results may not be generalizable or useful for targeting interventions in other populations, and do not capture any changes that have occurred in the correlates of beef consumption since the COVID-19 pandemic. At this point in time, however, post-pandemic NHANES dietary data are not available.

Individuals make dietary choices for lots of reasons, including taste, cultural upbringing, and socioeconomic conditions, which likely all rank ahead of health and nutrition concerns [31]. Motivations about the environment or animal welfare are even further down the list [28]. Interventions to address high consumption of beef or other foods with a high environmental impact could appeal to a variety of consumers’ values, potentially in combination. For example, red meat labels with combined health and environment warning messages could reduce purchases [32]. Other researchers have developed a taxonomy of programming that might lead to reductions in meat consumption. Such actions might either promote smaller portions (“less”), smaller portions using meat raised more sustainably (“less, but better”), smaller portions and eating more vegetable proteins (“less and more varied”), and meatless meals with or without meat substitutes (e.g., Meatless Monday) [33]. Extensions of this work should consider the full range of mixed dishes in which beef, or red meats, are consumed [30], and how to address common reasons for not reducing meat consumption, such as the perception that healthy meals include meat and that meals without meat are incomplete or boring [28].

Regardless of the approach, reducing beef consumption in the US would be beneficial to both health and the environment. Future work could examine US beef consumption over time, and whether this varies by demographic, socioeconomic, or behavioral factors. Enhancing dietary databases to allow for an easier disaggregation of beef, or other high-environmental-impact foods, would help to facilitate this work as well as other efforts to investigate dietary patterns and sustainability.

## 5. Conclusions

In conclusion, we used a novel approach to disaggregate beef from other red meat and to identify disproportionate beef consumers in a nationally representative US sample. One-eighth of consumers were identified as such, but their consumption accounted for half of total beef consumption for the day. Being male was the strongest predictor for disproportionate beef consumption in all models. This research can assist applied programming that seeks to reduce the climate impact of the food system by targeting awareness campaigns and educational programs towards those who consume the most beef.

## Figures and Tables

**Table 4:** Top 20 food types, ranked by the total amount of beef consumed by adults 18+ years, NHANES 2015–2018 (*n* = 10,238).

USDA Code	Food Category Description	Population-Level Consumption (Ounce Equivalents)			Respondents	
		Total	Percent of Total	Cumulative Percent	Frequency (*n*)	Frequency (%)
2002	Beef, excludes ground	106,781,150	30.7%	30.7%	1027	9.4%
3702	Burgers (single code)	62,882,785	18.1%	48.7%	947	9.4%
3002	Meat mixed dishes	50,067,281	14.4%	63.1%	796	8.1%
3502	Burritos and tacos	20,094,415	5.8%	68.9%	718	5.9%
2602	Cold cuts and cured meats	19,622,145	5.6%	74.5%	531	5.2%
2004	Ground beef	15,698,913	4.5%	79.0%	184	2.0%
3703	Frankfurter sandwiches (single code)	14,148,461	4.1%	83.1%	330	3.3%
2608	Sausages	7,945,033	2.3%	85.4%	255	2.2%
3802	Soups	7,175,849	2.1%	87.4%	257	1.6%
3204	Pasta mixed dishes, excludes macaroni and cheese	6,616,863	1.9%	89.3%	434	4.3%
3708	Other sandwiches (single code)	6,479,862	1.9%	91.2%	130	1.5%
3602	Pizza	6,129,408	1.8%	93.0%	891	7.6%
3404	Stir fry and soy-based sauce mixtures	4,735,840	1.4%	94.3%	81	0.7%
3506	Other Mexican mixed dishes	3,902,302	1.1%	95.4%	186	1.5%
3706	Egg/breakfast sandwiches (single code)	3,298,461	0.9%	96.4%	281	2.6%
2606	Frankfurters	3,156,976	0.9%	97.3%	84	0.6%
3504	Nachos	1,462,181	0.4%	97.7%	49	0.4%
3206	Macaroni and cheese	1,321,655	0.4%	98.1%	16	0.2%
3402	Fried rice and lo/chow mein	1,254,718	0.4%	98.4%	30	0.3%
3208	Turnovers and other grain-based items	1,167,858	0.3%	98.8%	91	1.0%

**Notes:** Food groups are based on the USDA’s WWEIA (What We Eat in America) Food Categories. Data are from adults aged 18+ years with reliable day 1 diet recalls in the 2015–2018 NHANES (*n* = 10,248, representing a population of 244,067,443). Ounce-equivalents are the unit used in the USDA Food Patterns Equivalents Database and represent the lean fraction of protein-rich foods. Additional fat from non-lean beef is allocated to the FPED solid fats category and is not represented here.

## Data Availability

Data from the NHANES can be found at https://www.cdc.gov/nchs/nhanes/index.htm. The FPED is available at https://www.ars.usda.gov/northeast-area/beltsville-md-bhnrc/beltsville-human-nutrition-research-center/food-surveys-research-group/docs/fped-overview/.

## Supplementary Materials

The following supporting information can be downloaded at: https://www.mdpi.com/article/10.3390/nu15173795/s1, Table S1: Percent of total ounce-equivalents of beef consumption from top 20 food categories, by age and gender, NHANES 2015–2018 (*n* = 10,238).
