# Supplementary material for: Demographic and Socioeconomic Correlates of Disproportionate Beef Consumption among US Adults in an Age of Global Warming
Source: Nutrients. 2023 Aug 30;15(17):3795. doi: 10.3390/nu15173795 (PMC10489941; doi:10.3390/nu15173795)
Supplement: Supplementary file 1 [file nutrients-15-03795-s001.zip › nutrients-2521619-SI.pdf]

Supplemental Table S1. Percent of total ounce-equivalents of beef consumption from top 20 food categories, by age and gender, NHANES 2015-2018 (N=10,238)

USDA Code	Food category description	Females				Males			
		18-29y N=1044	30-49y N=1668	50-65y N=1442	66+ y N=1125	18-29y N=974	30-49y N=1457	50-65y N=1400	66+ y N=1125
2002	Beef, excludes ground	27.2%	26.6%	26.9%	22.6%	25.4%	39.8%	35.8%	23.2%
3702	Burgers (single code)	19.8%	18.0%	15.5%	10.1%	23.4%	19.5%	15.5%	19.4%
3002	Meat mixed dishes	14.4%	15.9%	19.4%	26.4%	13.6%	7.4%	13.1%	19.0%
3502	Burritos and tacos	9.5%	8.9%	3.2%	5.6%	8.2%	5.5%	3.9%	2.7%
2602	Cold cuts and cured meats	4.7%	3.6%	5.2%	7.8%	5.8%	4.1%	7.4%	8.7%
2004	Ground beef	1.5%	3.5%	9.6%	6.1%	3.7%	2.3%	5.1%	6.2%
3703	Frankfurter sandwiches (single code)	3.6%	4.7%	2.3%	4.5%	5.1%	4.4%	3.5%	4.3%
2608	Sausages	2.3%	4.1%	1.9%	1.2%	1.6%	1.9%	1.6%	4.7%
3802	Soups	0.9%	3.2%	3.6%	2.7%	0.5%	2.6%	1.1%	1.9%
3204	Pasta mixed dishes, excludes macaroni and cheese	2.3%	1.7%	2.2%	2.3%	1.8%	1.3%	2.3%	2.2%
3708	Other sandwiches (single code)	1.0%	1.4%	1.0%	1.7%	2.3%	1.6%	2.9%	2.4%
3602	Pizza	2.8%	1.8%	1.0%	1.1%	2.3%	2.1%	1.6%	0.7%
3404	Stir-fry and soy-based sauce mixtures	2.3%	0.9%	1.4%	2.2%	1.2%	1.3%	1.5%	0.6%
3506	Other Mexican mixed dishes	1.3%	1.1%	2.5%	2.1%	0.8%	0.8%	0.7%	0.6%
3706	Egg/breakfast sandwiches (single code)	0.6%	1.3%	0.7%	0.8%	1.1%	1.3%	0.6%	0.7%
2606	Frankfurters	0.9%	0.7%	0.6%	1.2%	0.6%	1.3%	0.7%	1.7%
3504	Nachos	0.4%	0.5%	0.1%	0.0%	0.7%	0.9%	0.1%	0.0%
3206	Macaroni and cheese	1.9%	0.6%	0.1%	0.1%	0.4%	0.4%	0.0%	0.0%
3402	Fried rice and lo/chow mein	0.0%	0.1%	0.5%	0.0%	0.3%	0.4%	0.8%	0.0%
3208	Turnovers and other grain-based items	0.8%	0.2%	0.3%	0.4%	0.4%	0.4%	0.3%	0.1%
	**Percent of total beef consumption:**	98.1%	98.9%	97.9%	98.9%	99.1%	99.4%	98.4%	98.9%

Notes: Food groups are based on USDA's WWEIA (What We Eat in America) Food Categories. Data are from adults aged 18+ years with reliable day 1 diet recalls in the 2015-2018 NHANES (n=10,248, representing a population of 244,067,443). Ounce-equivalents are the unit used in the USDA Food Patterns Equivalents Database and represent the lean fraction of protein-rich foods. Additional fat from non-lean beef is allocated to the FPED solid fats category and is not represented here.
